# Prevalence and influencing factors of frailty in older patients with diabetes in China: a system review and meta-analysis

**DOI:** 10.3389/fmed.2023.1199203

**Published:** 2023-09-21

**Authors:** Jian Liu, Yanjun Cao, Qingjie Wang, Zhiwei Wang, Xiaorong Luan

**Affiliations:** ^1^School of Nursing and Rehabilitation, Cheeloo College of Medicine, Shandong University, Jinan, Shandong, China; ^2^Department of Neurology, The Second Hospital, Cheeloo College of Medicine, Shandong University, Jinan, Shandong, China; ^3^Endocrinology Department, Feicheng People's Hospital, Feicheng, Shandong, China; ^4^Department of Infection Control, Qilu Hospital, Shandong University, Jinan, Shandong, China

**Keywords:** The aged, diabetes mellitus, frailty, risk factor, China

## Abstract

**Background and aims:**

To systematically evaluate the relevant literature to explore the prevalence and influencing factors of frailty in older patients with diabetes in China.

**Methods:**

Cochrane Library, PubMed, Embase, Medline, CINAHL, Scopus, Proquest Central, Web of Science, SinoMed, CNKI, VIP and Wan fang Databases were searched to collect Chinese and English literatures about frailty in older diabetic patients. RevMan 5.4 software was used to extract data for systematic review.

**Results:**

Seventeen studies involving 23,070 older patients with diabetes were included. The results showed that the prevalence of frailty in older Chinese diabetic patients was 30%. The main influencing factors were HbA1c level, number of complications, age, depression, exercise, and nutritional status.

**Conclusion:**

The prevalence of frailty in Chinese elderly diabetic patients is high and there are many influencing factors. However, the quality of relevant literature is general and the number is limited, so high-quality prospective studies should be carried out in the future to further verify the conclusions.

## 1. Introduction

China has the highest number of older diabetics in the world, with a prevalence of diabetes of 20.2% among those aged 60 years or older (1). Older people with diabetes have a 5-fold increased risk of frailty than non-diabetic patients (2). This study used the concept of physical frailty proposed by Fried et al. in 2004 (3). Physical Frailty is a syndrome of old age in which decreased physiological reserve leads to increased vulnerability of the body (3), which leads to decreased mobility (4) and increased difficulty in monitoring and managing blood glucose in older diabetics (5). As current research continues to deepen, more scholars have categorized physical frailty into pre-physical frailty and physical frailty, and Sezgin et al. defined pre-physical frailty as a complex, multifactorial, and multidimensional state related to the progression of physical impairments over time through a systematic review and qualitative analysis approach, and as a transitory and potentially reversible state of risk prior to physical frailty (6); the concept of physical frailty As mentioned above. Different subtypes have been elaborated for the concept of physical frailty, such as cognitive frailty, emotional frailty, etc., but these subtype concepts are usually combinations of physical frailty, distinguishing them from the current subtype concepts regarding physical frailty, which this scholar considers to occur prior to the individual frailty subtypes.

The mechanism of its occurrence can be explained by the fact that diabetes impairs vascular function and accelerates the reduction of skeletal muscle, which leads to increased debility (7). The current status of frailty in older diabetic patients has been investigated both nationally and internationally, but the results of various studies (4, 8, 9) have been mixed. Kong et al. (10) were the first to provide a systematic review of the current status of frailty in older diabetic patients, but the study included older adults from different countries in the community, which may lead to a high level of heterogeneity. Gao et al. (11) conducted a systematic evaluation of the current status of frailty in older diabetic patients originating from different locations, but this study only compared the regional differences in the prevalence of frailty and did not address the influencing factors. An objective understanding of the current status of debilitation in older diabetic patients in China and clarification of its influencing factors would be beneficial for health care professionals to identify indicators of sensitivity in high-risk groups. Therefore, this study is a comprehensive collection of studies on debilitation in older diabetic patients in China, aiming to obtain findings with some reference value for health care professionals to screen high-risk groups and develop debilitation prevention measures.

## 2. Materials and methods

### 2.1. Inclusion criteria

(1) Study population: the definition of older persons may vary in different countries and regions, but is usually based on age and related characteristics. This study was conducted in the Chinese context, so we set the diabetic patients ≥60 years old; (2) study content: assessment tools for frailty must be explicitly mentioned in the literature; (3) outcome indicators: prevalence of frailty and influencing factors; (4) study type: cross-sectional studies, cohort studies, and retrospective studies, language limited to Chinese and English.

### 2.2. Exclusion criteria

(1) Only abstracts were published or full text was not available; (2) data in the original study could not be converted and applied; (3) duplicate published literature; (4) debilitation combined with specific disease populations.

### 2.3. Literature search strategy

Cochrane Library, PubMed, Embase, Medline, CINAHL, Scopus, Proquest Central, Web of Science, SinoMed, CNKI, VIP and Wan fang Databases were conducted. Searches were performed with a combination of subject terms and free words and retrospectively incorporated into the literature. The search time frame was from the establishment of the database to August 2023. The search terms are: (“aged” OR “elder*”) AND (“diabetes mellitus” OR “diabetes distress”) AND (“frail*” OR “frailty syndrome” OR “weakness”) AND (“influence factor*” OR “risk factor*”).

### 2.4. Literature screening and data extraction

Literature screening and data extraction were cross-checked by 2 investigators independently according to the inclusion and exclusion criteria of the literature. In case of disagreement, a third researcher was consulted through discussion or to assist in judgment. Data extraction included: author, year of publication, region of investigation, source of study population, sample size, age, debilitating assessment tool, number of debilitating individuals, and influencing factors.

### 2.5. Literature quality evaluation

The quality evaluation of the included literature was performed by 2 researchers, and in case of disagreement, the decision was made through consultation with a third researcher. The quality evaluation of cross-sectional studies was based on the quality evaluation tool of The Joanna Briggs Institute (JBI), an Australian evidence-based health care center (12), with 9 evaluation entries, all categorized as “yes” “no” “unclear” and “not applicable” were evaluated in 4 levels, and 9 criteria were fully satisfied as level A, which is low bias; partially satisfied as level B, which is moderate bias; and 1 or more criteria were not satisfied as level C, which is high bias.

### 2.6. Statistical methods

RevMan 5.4 software was used for statistical analysis, and the combined effect size was expressed as the ratio of ratios (OR) and 95%CI, and the combined data were tested for heterogeneity and combined with *I^2^* to evaluate the magnitude of heterogeneity. If *P* > 0.10 and *I^2^* ≤ 50%, the studies were homogeneous and a fixed-effects model was selected for systematic evaluation; if *P* ≤ 0.10 and *I^2^*>50%, the studies were heterogeneous and a random-effects model was selected for systematic evaluation. Subgroup analysis was performed according to possible sources of heterogeneity to explore and reduce heterogeneity, and sensitivity analysis was used to evaluate the stability of the results. Differences were considered statistically significant at *P* < 0.05. Funnel plots were used to determine whether there was publication bias in the included literature.

## 3. Results

### 3.1. Literature search results

**Figure 1:**
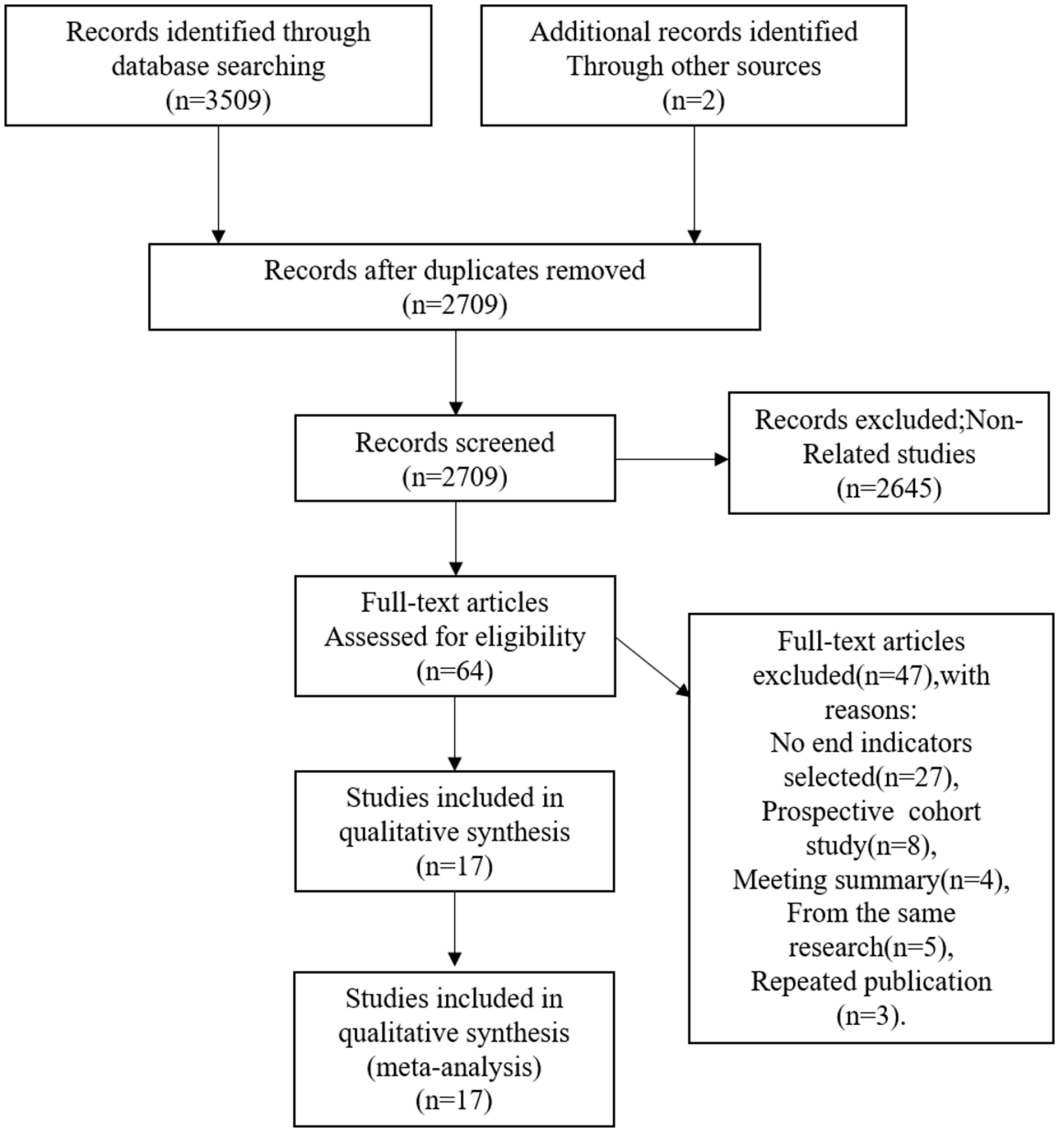
Flow diagram for identifying studies.

A total of 2,709 publications (2,443 in English and 266 in Chinese) were obtained after the search. After screening according to the inclusion and exclusion criteria, 17 papers (4, 8, 9, 13–26) were finally included, including 2 in English and 15 in Chinese, as shown in Figure 1.

### 3.2. Basic characteristics and quality assessment of the included literature

A total of 17 cross-sectional studies were included in this study, 13 in the literature with hospital patients and 4 in the literature with community populations. The basic characteristics and methodological quality evaluation of the included literature are shown in Table 1.

**Table 1:** Basic characteristics of the included literature and evaluation of methodological quality.

Author and year of publication	Survey area	Source of research subjects	Number of diabetic patients (cases)	Age (years)	Survey tools	Number of debilitation (cases)	Influencing factors	Quality evaluation
Kong et al. (4), 2021	Hubei Xianning	Community	291	≥65	①	56	7, 9, 13, 15	B
Wu et al. (15), 2021	Hunan Hengyang	Community	254	≥60	①	70	7, 8, 14, 16	B
Ge1 et al. (17),2020	Liaoning Shenyang	Hospital	221	≥65	②	137	1, 4, 10, 14	B
Cheng et al. (16),2020	Tianjin	Hospital	998	≥60	③	80	8, 9, 10, 15	B
Yang et al. (19),2020	Hunan Hengyang	Community	343	≥60	④	198	3, 5, 8, 9, 10, 17	A
Ge2 et al. (18),2020	Anhui Hefei	Hospital	251	≥60	①	54	2, 9, 10	B
Zhao et al. (20),2019	Xinjiang Uygur Autonomous Region	Hospital	431	≥60	①	144	1, 2, 9, 10, 14, 15	B
Chen2 et al. (14),2019	Ningxia Hui Autonomous Region	Hospital	278	≥60	①	41	1, 4, 8, 9, 10, 11	B
Jia et al. (8), 2019	Henan Zhengzhou	Hospital	296	≥65	④	137	3, 6, 9, 10, 16	B
Guo et al. (13), 2019	Sichuan Chengdu	Hospital	310	≥60	①	167	1, 9, 10, 13	B
Chen1 et al. (9), 2019	Beijing	Hospital	126	≥65	③	46	13, 14, 17	B
Long et al. (21), 2023	Sichuan Yibin	Hospital	360	≥60	③	52	1, 10, 13, 14	B
Zhang et al. (22), 2023	Beijing	Hospital	343	≥65	③	108	18	B
Zhang1 et al. (23), 2022	Chongqing	Hospital	135	≥60	③	49	1, 3, 9, 10, 13, 14	B
Bao et al. (24), 2022	Jiangsu	Hospital	210	≥65	⑤	63	2, 18	B
Zhang2 et al. (25), 2022	Shanxi Xian	Hospital	213	≥60	③	65	1, 13, 14, 15	B
Zeng et al. (26), 2022	Random sample in mainland China	Community	18,010	≥60	⑥	4,034	10, 12, 14	A

① Fried debilitation phenotype scale; ② Chinese version of Edmonton debilitation scale; ③ FRAIL debilitation scale; ④ Tilburg debilitation scale; ⑤ Comprehensive geriatric assessment; ⑥ Frailty Index.

Influencing factors: 1. age; 2. gender; 3. education level; 4. economic status; 5. marital status; 6. smoking; 7. alcohol consumption; 8. exercise time; 9. glycosylated hemoglobin; 10. other comorbid diseases; 11. hospitalization frequency; 12. economic status; 13. nutrit ional status; 14. ability to perform activities of daily living; 15. depression; 16. self management level; 17. cognition Status; 18. grip.

Influencing factors: (1) age; (2) gender; (3) education level; (4) economic status; (5) marital status; (6) smoking; (7) alcohol consumption; (8) exercise time; (9) glycosylated hemoglobin; (10) other comorbid diseases; (11) hospitalization frequency; (12) economic status; (13) nutritional status; (14) ability to perform activities of daily living; (15) depression; (16) self-management level; (17) cognition Status; (18) grip.

### 3.3. Systematic evaluation of the prevalence of frailty in older patients with diabetes mellitus

**Figure 2:**
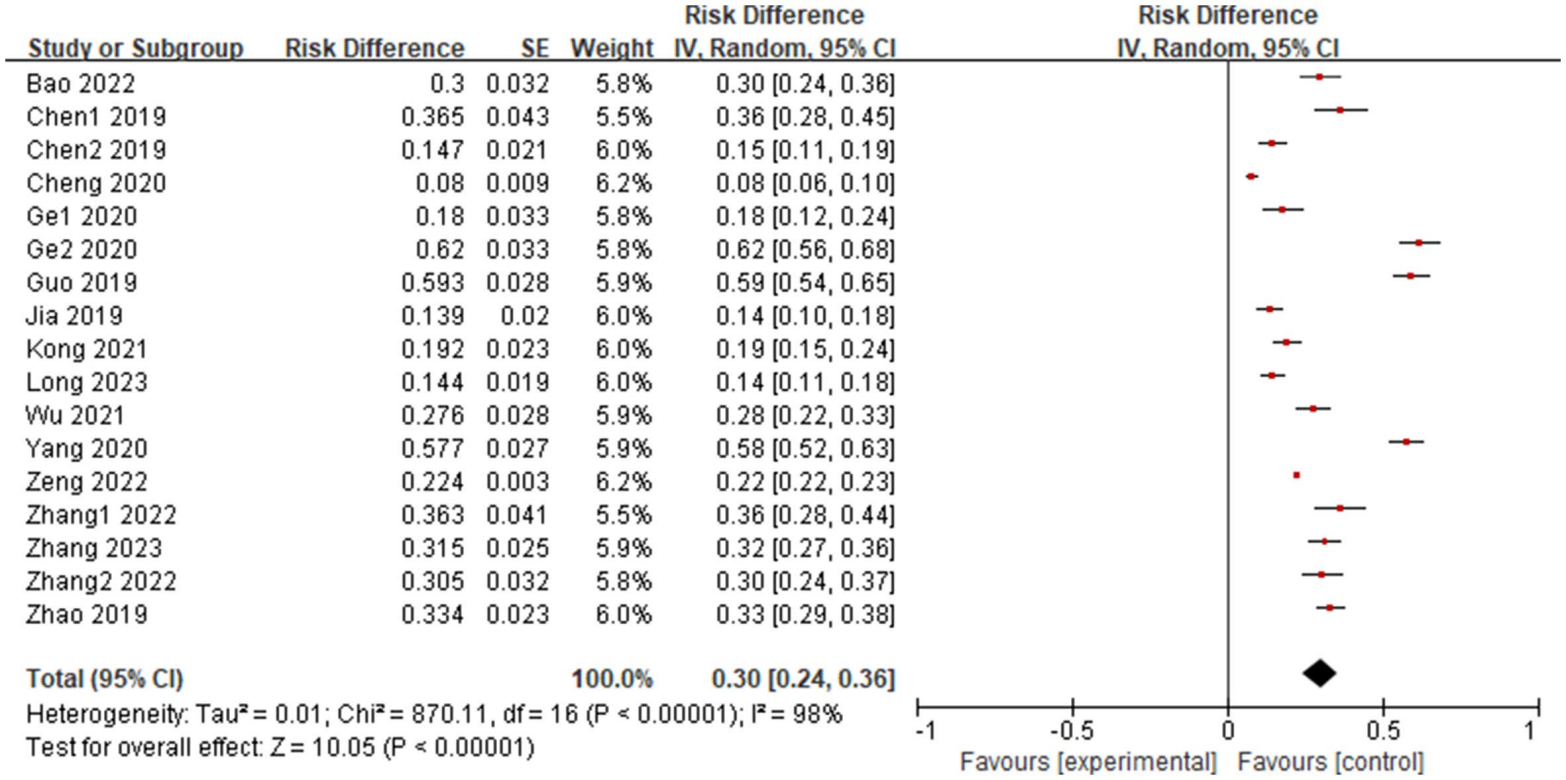
Systematic evaluation of the prevalence of debilitation in older diabetic patients in China.

Seventeen (4, 8, 9, 13–26) cross-sectional studies with a total of 23,070 older diabetic patients were systematically evaluated for the prevalence of frailty. Because of the high heterogeneity among the included studies (*I^2^* = 98%, *P* < 0.01), a random-effects model was used. The results showed that the prevalence of debilitation in Chinese older diabetic patients was 30% (95%*CI*, 0.24 ~ 0.36, *P* < 0.01), and the combined effect was statistically significant, as shown in Figure 2.

### 3.4. Subgroup analysis of frailty prevalence in older diabetic patients

Due to the high heterogeneity among the included studies, this study was subgroup analyzed by gender, source of study population, and frailty assessment tool. The results of the subgroup analysis showed that the prevalence of frailty in older diabetic patients was higher in women (32%) than in men (25%); the prevalence of frailty in older diabetic patients was higher in Community (32%) than in hospitals (30%); and the prevalence of frailty in older diabetic patients was higher when measured by the Tilburg frailty scale (36%) than by the Fried frailty phenotype scale (28%) and the FRAIL frailty scale (26%), see Table 2.

**Table 2:** Results of subgroup analysis of the prevalence of frailty in older diabetic patients.

	Combined effect volume		Heterogeneity test	
Grouping criteria	Number of studies	OR(95%CI)	*P*-value	*I*^2^-value (%)
Gender				
Male	12	0.25 (0.17,0.34)	<0.01	96
Female	12	0.32 (0.21,0.42)	<0.01	97
Source of study subjects				
Hospital	13	0.30 (0.21,0.39)	<0.01	98
Community	4	0.32 (0.18,0.45)	<0.01	98
Frailty assessment tools				
Fried Frailty Phenotype Inventory	6	0.28 (0.18,0.39)	<0.01	97
FRAIL Frailty Scale	6	0.26 (0.15,0.37)	<0.01	97
Tilburg Frailty Scale	2	0.36(−0.07,0.79)	<0.01	99

### 3.5. Systematic evaluation of factors influencing the occurrence of debilitation in older diabetic patients

The six influencing factors were systematically evaluated, and because of the high heterogeneity of the influencing factors among studies, a random-effects model was used for the analysis, and the results showed that the combined *OR* and 95% *CI* of each influencing factor were statistically significant, as shown in Table 3.

**Table 3:** Results of meta-analysis of factors influencing the occurrence of debilitation in older diabetic patients.

Influencing factors	Inclusion in the literature	Heterogeneity test		Combined effect volume		*Z*-value	*P*-value
		*P*-value	*I^2^*	*OR*	95%*CI*		
Glycated hemoglobin level	8	<0.01	99	0.31	0.17 ~ 0.44	4.55	*P* < 0.01
Number of complications	10	<0.01	99	0.32	0.23 ~ 0.40	7.43	*P* < 0.01
Age	7	<0.01	97	0.22	0.10 ~ 0.33	4.78	*P* < 0.01
Depression status	5	<0.01	97	0.30	0.10 ~ 0.51	3.77	*P* = 0.0002
Sports and exercise	4	<0.01	98	0.33	0.16 ~ 0.51	3.72	*P* = 0.0002
Nutritional status	6	<0.01	97	0.33	0.18 ~ 0.47	4.46	*P* < 0.01

### 3.6. Sensitivity analysis and publication bias

Sensitivity analysis was performed after excluding each of the 17 cross-sectional studies in which the outcome indicator was the prevalence of debilitation, and no significant change in the combined effect size was observed, as shown in Table 4. In addition, funnel plots drawn with studies reporting the prevalence of debilitation showed that the effect points of each study were approximately symmetrically distributed with the combined effect size at the center. Because the gray literature was not included in this study and the number of included studies was small, publication bias cannot be completely ruled out.

**Table 4:** Results of sensitivity analysis for systematic evaluation of the current status and influencing factors of frailty in older diabetic patients.

Excluding the first author of the study and the year of publication	Combined effect size after exclusion [*OR* (95%*CI*)]
Cheng, 2020 (16)	0.32 [0.25, 0.38]
Jia, 2019 (8)	0.31 [0.25, 0.37]
Chen2, 2019 (14)	0.31 [0.25, 0.37]
Kong, 2021 (4)	0.31 [0.25, 0.37]
Ge2, 2020 (18)	0.28 [0.23, 0.34]
Wu, 2021 (15)	0.30 [0.24, 0.36]
Zhao, 2019 (20)	0.30 [0.24, 0.36]
Chen1, 2019 (9)	0.30 [0.24, 0.36]
Guo, 2019 (13)	0.28 [0.23, 0.34]
Yang, 2020 (19)	0.28 [0.23, 0.34]
Long, 2023 (21)	0.31 [0.25, 0.37]
Zhang, 2023 (22)	0.30 [0.24, 0.36]
Zhang1, 2022 (23)	0.30 [0.24, 0.36]
Zhang2, 2022 (25)	0.30 [0.24, 0.36]
Bao, 2022 (24)	0.30 [0.24, 0.36]
Zeng, 2022 (26)	0.31 [0.22, 0.39]
Ge1, 2020 (17)	0.31 [0.25, 0.37]

## 4. Discussion

### 4.1. High prevalence of debilitation in older diabetic patients in China

The results of this study showed that the prevalence of debilitation in older diabetic patients in both Community (32%) and hospital (30%) in China was higher than the systematic evaluation by Peng et al. of the prevalence of debilitation in older people in Community (12.8%) and hospital (22.6%) in China. All three communities were studied with convenience sampling. It may be related to the fact that diabetes leads to endocrine metabolic disorders and increased inflammatory response in the older, resulting in a higher prevalence of debilitation in older diabetic patients (7). In addition, the different choices of the three scales used more often in this study contributed to the differences that existed between the studies. The Frailty Phenotype Scale is defined as unexplained weight loss, low grip strength, fatigue, slow walking speed, and decreased physical activity, with more than three items defined as frailty; the FRAIL Scale includes fatigue, endurance, mobility, illness, and weight loss in the last year, with more than three items defined as frailty; and the Frailty Assessment Scale for the Elderly Scale Score calculates a scale score by dividing the cumulative score of the individual’s deficient entry by the total number of entries. Score. Scores range from 0 to 1, with higher scores indicating greater frailty. The results suggest that frailty is a serious problem in older diabetic patients in hospitals and communities, and health care workers need to conduct early screening for frailty, detect it early and give interventions to avoid the occurrence of adverse health outcomes in patients.

### 4.2. Factors influencing the occurrence of frailty in older diabetic patients in China

#### 4.2.1. Glycosylated hemoglobin level

The results of this study showed that glycosylated hemoglobin level was an influencing factor for frailty in older diabetic patients. Zaslavsky et al. (27) found a U-shaped relationship between blood glucose levels and frailty in older diabetic patients, i.e., minimum and maximum blood glucose levels were associated with an increased risk of frailty. This suggests that clinical care providers need to determine the optimal level of glycemic control for older diabetic patients to reduce the prevalence of frailty.

#### 4.2.2. Number of comorbidities

The high number of complications is a risk factor for frailty in older diabetic patients. Castrejon et al. (28) showed that any diabetic complication is significantly associated with frailty, which is consistent with the results of this study. The possible reason is that diabetic complications lead to increased vulnerability of organs in the body. in addition, the coexistence of multiple diseases requires multiple medications, and adverse reactions between medications can accelerate the decline of body functions. Therefore, health care staff should provide the best treatment plan and care measures to delay the onset of diabetic complications.

#### 4.2.3. Age

Advanced age is a risk factor for debilitation in older diabetic patients. This is consistent with previous studies (4, 29) and may be due to the fact that with increasing age, older diabetic patients experience metabolic disorders and degeneration of various body organ systems, which can be triggered by smaller events. Studies (30) have shown that the likelihood of debilitation increases by 10% with each additional year of age. Therefore, the assessment of older diabetic patients should be enhanced in clinical care.

#### 4.2.4. Depressive states

This study found a higher prevalence of debilitation in older diabetic patients with depressive states. Previous evidence (31) suggests that there is an interaction between depression and frailty in older diabetic patients, with the possible cause being physical frailty leading to functional dependence or disability, which leads to depression. Therefore, caregivers need to manage the depressive state of older diabetic patients and provide timely psychological care interventions to delay the deterioration of physical debilitation in this population.

#### 4.2.5. Exercise

Exercise and movement can reduce the occurrence of debilitation in older diabetic patients. Some studies have shown (32) that exercise exercise is a protective factor for combined debilitation in older diabetic patients. The possible reason is that reasonable exercise exercise helps to improve the body’s sensitivity to insulin (19) improve inflammation and oxidative stress (15), and thus enhance the body’s energy reserves. Therefore, health care professionals should enhance the intervention of exercise training for patients while ensuring their safety, thus maintaining the functional state of the body and reducing the occurrence of debilitation.

#### 4.2.6. Nutritional status

The results of this study showed that malnutrition was associated with a high prevalence of frailty in older diabetic patients. Cruz et al. (33) indicated that malnutrition is chronically prevalent in diabetic patients, making this population more prone to frailty. Possible reasons for this are aging leading to decreased appetite and limited activity in older diabetic patients (9). This suggests that health care providers should screen the nutritional status of older diabetic patients in a timely manner and provide them with appropriate dietary guidance would be an effective way to prevent the onset of debilitation in this population.

## 5. Limitations

Only Chinese and English literature was included in this study, and there may be language bias; the final included were cross-sectional studies, and the results still need further validation; the heterogeneity among studies was high, and although this study attempted a subgroup analysis of possible sources of heterogeneity, no specific source was found, and it is speculated that it may be that current debilitating assessment tools are not uniform; In the future, we will analyze the pre-frailty of diabetes mellitus as an important variable to better guide clinical research.

## 6. Conclusion

This study found a high prevalence of frailty in current Chinese older diabetic patients with a number of influencing factors. Facing the older diabetic debilitated patients in hospitals and Community, health care workers should strengthen the monitoring of factors such as blood glucose level and psychological problems, and make effective interventions, which can reduce the occurrence of debilitation to some extent. Currently, most of the older diabetic patients are focused on the current situation investigation, and it is expected that future high-quality cohort studies will be conducted to verify the causal relationship, and future studies should also pay attention to the pre-frailty status of older diabetic patients.

## Data availability statement

The raw data supporting the conclusions of this article will be made available by the authors, without undue reservation.

## Author contributions

JL, YC, and XL designed the research. JL, QW, and ZW collected the data. JL, QW, and YC analyzed the data. JL and YC wrote the manuscript. YC, QW, and ZW reviewed and edited the manuscript. XL had primary responsibility for final content. All authors have read and agreed to the published version of the manuscript.

## Funding

This study was sponsored by the Natural Science Foundation of Shandong Province (no. ZR2020MG071).

## Conflict of interest

The authors declare that the research was conducted in the absence of any commercial or financial relationships that could be construed as a potential conflict of interest.

## Publisher’s note

All claims expressed in this article are solely those of the authors and do not necessarily represent those of their affiliated organizations, or those of the publisher, the editors and the reviewers. Any product that may be evaluated in this article, or claim that may be made by its manufacturer, is not guaranteed or endorsed by the publisher.
